# Role of the *Mycoplasma pneumoniae*/Interleukin-8/Neutrophil Axis in the Pathogenesis of Pneumonia

**DOI:** 10.1371/journal.pone.0146377

**Published:** 2016-01-11

**Authors:** Zhengrong Chen, Xuejun Shao, Xunwu Dou, Xinxing Zhang, Yuqing Wang, Canhong Zhu, Chuangli Hao, Mingyue Fan, Wei Ji, Yongdong Yan

**Affiliations:** 1 Department of Respiratory Disease, Children's Hospital of Soochow University, Soochow University, Suzhou, China; 2 Department of Clinical Laboratory, Children's Hospital of Soochow University, Soochow University, Suzhou, China; 3 Department of Ophthalmology and Otorhinolaryngology, Children's Hospital of Soochow University, Soochow University, Suzhou, China; Singapore Immunology Network, SINGAPORE

## Abstract

Neutrophil infiltration is the characteristic pathological feature of *M*. *pneumoniae* pneumonia (MPP). This study aimed to explore the associations among neutrophil activity, clinical presentation, and role of the *M*. *pneumoniae*/interleukin-8 (IL-8)/neutrophil axis in the pathogenesis of MPP. A total of 42 patients with MPP were prospectively enrolled in the study. Neutrophil activity, including matrix metalloproteinase-9 (MMP-9), myeloperoxidase (MPO), and neutrophil elastase (NE), were measured. Clinical information was collected for all patients and control group. *In vitro*, IL-8 production was measured at different time points after *M*. *pneumoniae* infection of bronchial epithelial cells, and neutrophil activity was analyzed after IL-8 stimulation. The percentage of neutrophil in the bronchoalveolar lavage fluid was higher in the group of patients with high levels of *M*. *pneumoniae* DNA than in those with low levels of *M*. *pneumoniae* DNA (P < 0.05). IL-8, MMP-9, and NE in patients with MPP significantly increased compared with controls and decreased after treatment (P < 0.05). MPO and MMP-9 were associated with duration of fever (r = 0.332, P < 0.05) and length of stay (r = 0.342, P < 0.05), respectively. *In vitro*, *M*. *pneumoniae* induced IL-8 production by bronchial epithelial cells in a time dependent manner. MPO, MMP-9 and NE production by neutrophils significantly increased compared with medium controls after IL-8 stimulation. In summary, the *M*. *pneumoniae*/IL-8/neutrophil axis likely plays a vital role in the pathogenesis of MPP.

## Introduction

Community-acquired pneumonia (CAP) is a major health problem in the world and is associated with substantial morbidity, mortality, and healthcare costs. Among all the infectious pathogens, *Mycoplasma pneumoniae* (*M*. *pneumoniae*) is one of the most important agents causing severe respiratory disease in children [1], accounting for up to 40% of CAP cases in children. Furthermore, as many as 18% of pediatric patients with *M*. *pneumoniae* pneumonia (MPP) require hospital admission [2].

The severity of MPP seems to depend on the host immune response to the infection through various mechanisms, including an allergic reaction to *M*. *pneumoniae*, *M*. *pneumoniae* virulence, host defenses, and polarization toward T-helper cell 1 or T-helper cell 2 predominance [3]. The characteristic pathological feature of human MPP is marked by lymphocytic infiltration in the peribronchovascular areas and accumulation of neutrophils and lymphocytes in the lung alveolar spaces. Neutrophils are sentinel cells of the innate immune system and are the principal cellular responders to acute inflammation [4]. Interleukin 8 (IL-8) production by bronchial epithelial cells can be induced by *M*. *pneumoniae* antigen or live *M*. *pneumoniae*, which then chemoattracts and activates neutrophils [5]. During acute pahse of MPP infection, the majority cells in bronchoalveolar lavage fluid of hamster are neutrophils which are replaced by lymphocytes in later phase of infection [6].

The present study aimed to explore the associations between neutrophil activity, clinical presentation and the role of the *M*. *pneumoniae/*IL-8/neutrophil axis in the pathogenesis of MPP in children who had been admitted to hospital. Our study also examined the process of IL-8 secretion by normal human bronchial epithelial (NHBE) cells and the activity of neutrophils stimulated by IL-8 in vitro.

## Materials and Methods

### Subjects

This study was approved by the Institutional Human Ethical Committee of the Children’s Hospital of Soochow University. Written consent was obtained from the guardians on behalf of the patients enrolled in this study. The patients were defined as MPP infection at following criteria: 1) Children with clinical symptoms of pneumonia (cough, fever, tachypnea, chest retractions, or abnormal auscultatory findings), confirmed by radiography and 2) *M*. *pneumoniae* DNA was detected in BALF by real-time polymerase chain reaction (PCR) and specific IgM and IgG antibodies against *M*. *pneumoniae* in paired sera were detected by enzyme-linked immunosorbent assays (ELISA). Patients were excluded if they were diagnosed as chronic lung disease, bronchopulmonary malformation, immunodeficiency, immunosuppression, cardiovascular disease, or were co-infected with other pathogens. Study was performed from January to December 2014 in 42 MPP patients. Fifteen of age matched control patients were selected from children who suffered from foreign body in the bronchus within 48 hours without secondary infection.

Demographic and clinical information were collected in all patients. Laboratory specimens were obtained including blood, nasopharyngeal aspirates (NPAs) and BALF. The following laboratory tests were conducted: C-reactive protein, alanine transaminase, L-lactate dehydrogenase and creatine kinase (type MB isoenzyme). Nine other viruses were detected by direct immunofluorescence assay and PCRs as previously described [7]. BALF cytology was also performed.

### BALF collection

Fiber optic bronchoscopy and BALF collection were performed as described previously [8]. BALF samples were examined for *M*. *pneumoniae* DNA, IL-8, matrix metalloproteinase 9 (MMP-9), myeloperoxidase (MPO) and neutrophil elastase (NE). Cells in BALF were counted based on Giemsa and Wright staining after centrifugation at 200 × g for 10 min at 4°C.

### Serology of *M*. *pneumoniae*

Specific IgM and IgG antibodies against *M*. *pneumoniae* were detected in serum samples of patients in the acute phase of MPP (on admission) and convalescent phase (on discharge) respectively, using a commercial ELISA kit (Serion ELISA classic *M*. *pneumoniae* IgG/IgM, Institute Virion/Serion, Würzburg, Germany) according to the manufacturer's instructions as previously described [9].

### Real-time PCR for *M*. *pneumoniae* detection

A real-time PCR procedure (Daan Gene Co. Ltd, Guangzhou, China) approved by the State Food and Drug Administration of China was used for the detection of *M*. *pneumoniae* as described previously [8]. In brief, one of the equally divided samples of BALF was shaken for 30 s and centrifuged at 15,000 × g for 5 min. The sediment was collected and DNA extracted from a 400 μl sample in accordance with the manufacturer’s instructions. Then, PCR amplification was conducted using primers and probes purchased from Daan Gene Company. Quantification curves were plotted using several concentrations of standard control samples.

### Examination of IL-8, MMP-9, MPO, and NE in BALF

The BALF samples were immediately centrifuged and preserved at -80°C for subsequent assays. IL-8, MMP-9, MPO and NE (R&D Company) levels in supernatant of BALF were measured by ELISA according to the manufacturer’s instructions.

### IL-8 secretion by NHBE cells infected with *M*. *pneumoniae in vitro*

The normal human bronchial epithelial (NHBE) cells were purchased from American Type Culture Collection (Bethesda, MD) and used at culture passages 3–5. The cells were grown in serum-free bronchial epithelial cell growth medium (BEGM; Clonetics, Houston TX) containing the following supplements (all from Clonetics): bovine pituitary extract (52 μg/ml), hydrocortisone (0.5 μg/ml), human epidermal growth factor (0.5 ng/ml), epinephrine (0.5 μg/ml), transferrin (10 μg/ml), insulin (5 μg/ml), retinoic acid (0.1 ng/ml), triiodothyronine (6.5 ng/ml), gentamycin (50 μg/ml), and amphotericinB (50 ng/ml).

The *M*. *pneumoniae* strain M129 was purchased from the Institute of Pathogen Biology, Medical College of University of South China. *M*. *pneumoniae* was grown in SP4 broth for 72 h at 37°C, spun at 10,000 × g for 20 min, re-suspended in saline to yield 1 × 10^8^ CFU/50μl and frozen at -80°C in aliquots that were subsequently used to infect epithelial cells. On the infection day, frozen *M*. *pneumoniae* aliquots were thawed, spun, resuspended in SP4 broth, and incubated for 2 h at 37°C. For infection with viable *M*. *pneumoniae*, the suspension of freshly harvested *M*. *pneumoniae* was diluted with supplement-free bronchial epithelial cell growth (BEGM) medium to obtain a designated infectious dose of 1–100 CFU/cell in six well plates (NHBE, 2×10^4^ cells/well in 2ml of serum-free BEGM). The supernatants were collected for IL-8 protein measurement by using an IL-8 ELISA kit (R&D Systems) at time points 2, 6, 12, 24, 48, and 72h.

### Release of MPO, MMP-9, and NE by neutrophils after stimulation with IL-8 or *M*. *pneumoniae in vitro*

Blood neutrophils were isolated from leucocyte-enriched buffy coats by Ficoll-Paque Plus gradient centrifugation and dextran sedimentation, as previously described [10]. Erythrocytes were removed by hypotonic lysis. The final cell pellet was suspended in RPMI-1640 medium (Sigma-Aldrich, Shanghai, China) supplemented with 50 U/ml penicillin, 50 μg/ml streptomycin to obtain 1×10^6^ cell/well. Cell viability was determined by frequency of cells without annexin V staining determined by flow cytometry analysis. More than 99% of the blood neutrophils were viable immediately before the culture assays. Isolated neutrophils were stimulated by IL-8 (10 ng/ml) for 24h. The supernatants of culture were collected and stored at -80°C. The levels of MPO, MMP-9 and NE released by neutrophils were measured using commercially available ELISAs as mentioned above.

### Data analysis

Numeration data were analyzed using the Chi-square test and measurement data were analyzed using the Student t-test or non-parametric test (Mann–Whitney U-test or Wilcoxon test) if the data distribution was non-normal. The Pearson or Spearman correlation test was used to assess correlations based on normal or abnormal distributed data. Associations between parameters and clinical profiles were analyzed using partial correlations. One-way analysis of variance (ANOVA) was used to identify differences between three or more groups. A two-sided p-value of < 0.05 was considered statistically significant. All analyses were performed using SPSS for Windows, version 17.0 software (SPSS Inc., Chicago, IL, USA).

## Results

### Demographic and clinical data of children with MPP

The demographic data, clinical presentation, and laboratory findings of the study patients with MPP were shown in Table 1. The mean age of control patients was 4.4 ± 2.4 years, and the male percentage was 60% (9/15). There was no statistical significance in age and gender between children with MPP and control subjects (both P > 0.05).

**Table 1:** Demographic and clinical profiles of study patients (children) with MPP.

Parameters	Patients with MPP
	n = 42
Age (mean ± SD, year)	5.6 ± 2.5
Male (n, %)	23 (54.8)
Duration of fever, (25th–75th percentile, d)	15.0 (13.0–18.0)
Length of stay, (mean ± SD, d)	10.9 ± 4.2
White blood cell count (mean ± SD, ×10^9^/L)	9.5 ± 4.8
Neutrophils (mean ± SD, ×10^9^/L)	6.8 ± 4.0
C-reactive protein (25th–75th percentile, mg/L)	20.9 (10.4–62.2)
Alanine transaminase increase (n, %)	8 (19.0)
l-lactate dehydrogenase (mean ± SD, U/L)	534.5 ± 227.3
MB isoenzyme of creatine kinase (25th–75th percentile, U/L)	16.4 (13.7–24.9)
Cytology of BALF (mean ± SD, %)	
Neutrophils	62.5 ± 23.1
Lymphocytes	8.9 ± 7.5
Macrophages	27.4 ± 22.2
Radiologic evaluation (n, %)	
Lobar or segmental opacity	42 (100)
Opacity with pleural effusion	13 (31.0)
Opacity with pulmonary atelectasis	3 (7.1)
Macrolide medication (n, %)	42 (100)
Methylprednisolone (n, %)	42 (100)

MPP: *Mycoplasma pneumoniae* pneumonia; BALF: bronchoalveolar lavage fluid; SD: standard deviation.

### Cytology and expressions of IL-8, MPO, MMP-9, and NE in BALF of children with MPP

**Fig 1:**
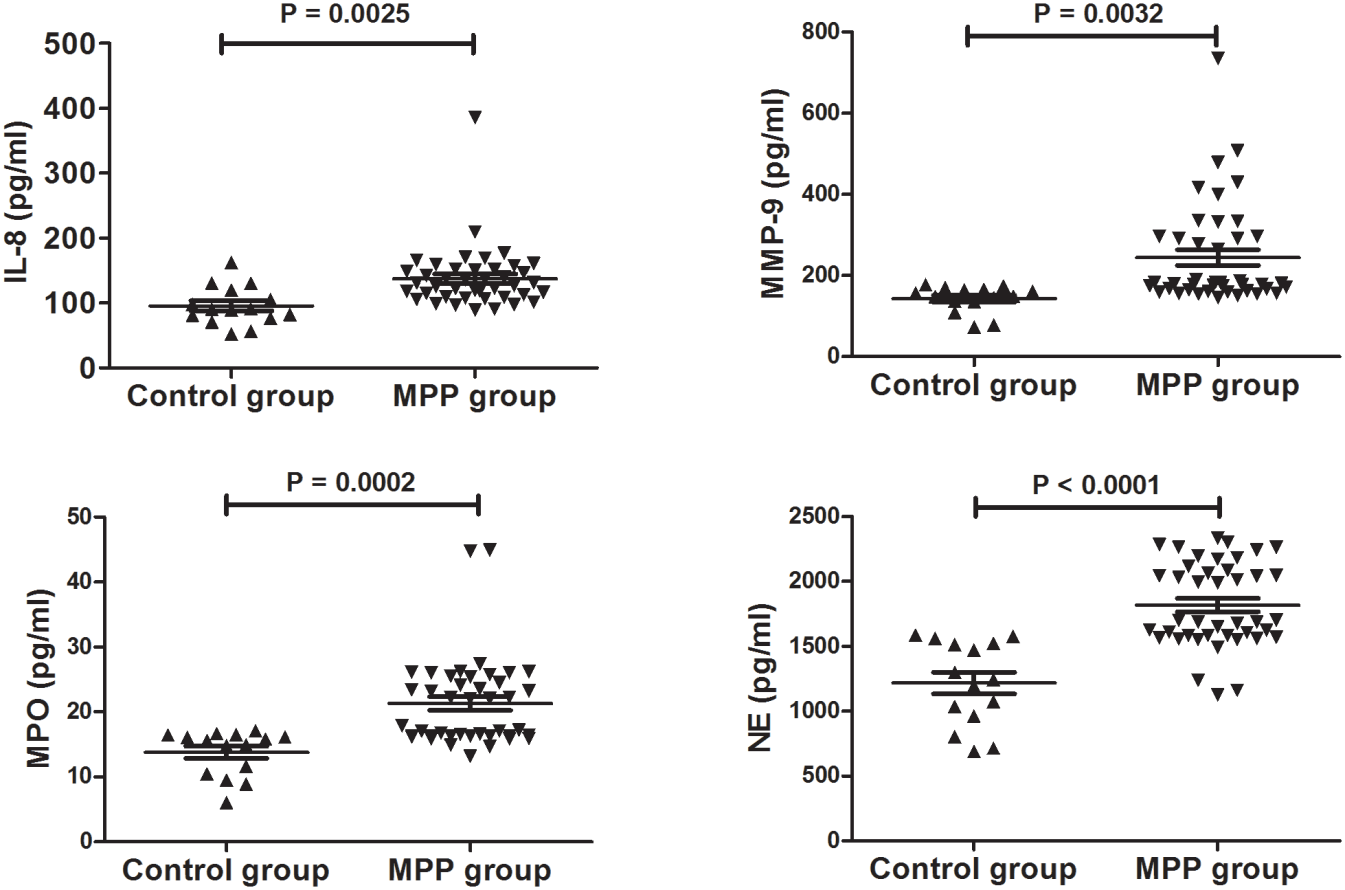
Comparison of IL-8, MPO, MMP-9, and NE levels in BALF between Patients with MPP and Controls. The values in the graphs represent the mean ± SD. The P values were calculated using Student’s t test while Mann-Whitney U-test was used for comparison of MMP-9 between MPP and control groups. MPP: *Mycoplasma pneumoniae* pneumonia; IL: interleukin-8; MPO: myeloperoxidase; MMP-9: matrix metalloproteinase 9; NE: neutrophil elastase.

**Fig 2:**
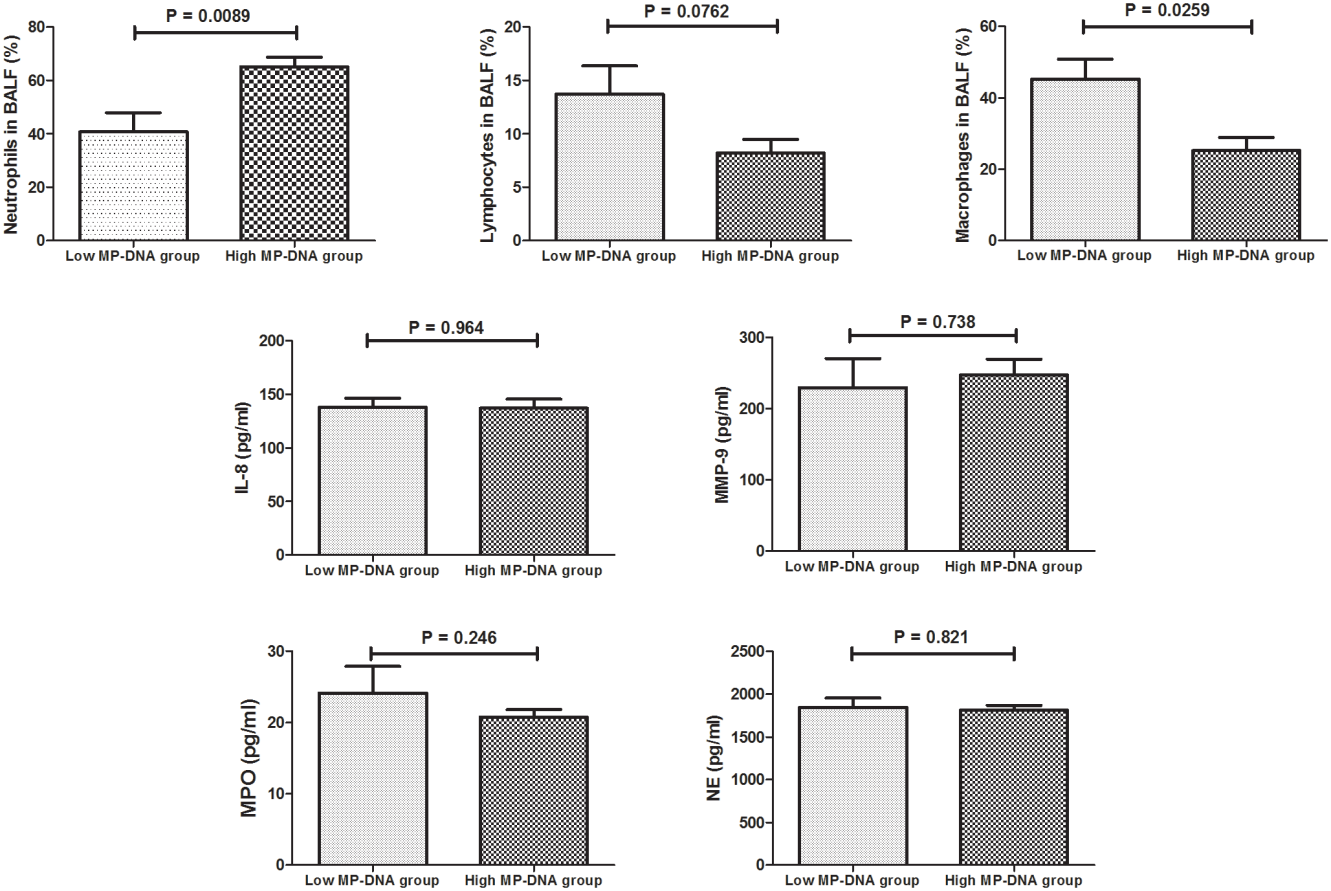
Comparison of Cytology, IL-8, MPO, MMP-9, and NE in BALF between the Low MP-DNA Group (< 10^7^ copies/ml) and High MP-DNA Group (≥ 10^7^ copies/ml). The values in the graphs represent the mean ± SD. The P values were calculated using Student’s t test while Mann-Whitney U-test was used for comparison of MMP-9 between two groups. MPP: *Mycoplasma pneumoniae* pneumonia; IL: interleukin-8; MPO: myeloperoxidase; MMP-9: matrix metalloproteinase 9; NE: neutrophil elastase.

As shown in Fig 1, the levels of IL-8, MPO, MMP-9, and NE in BALF in patients with MPP were significantly higher than in the controls. According to the concentration of *M*. *pneumoniae* DNA in BALF, all MPP cases were divided into a low *M*. *pneumoniae* DNA group (< 10^7^ copies/ml) and a high *M*. *pneumoniae* DNA group (≥10^7^ copies/ml). BALF neutrophil percentage was higher in the high *M*. *pneumoniae* DNA group than in the low *M*. *pneumoniae* DNA group (Fig 2). However, the low *M*. *pneumoniae* DNA group had a higher percentage of macrophages in the BALF than the high *M*. *pneumoniae* DNA group. No significant difference between the two groups was found for levels of IL-8, MPO, MMP-9, and NE (Fig 2).

### Comparisons of IL-8, MPO, MMP-9, and NE expressions in BALF between children with and without pleural effusion

**Fig 3:**
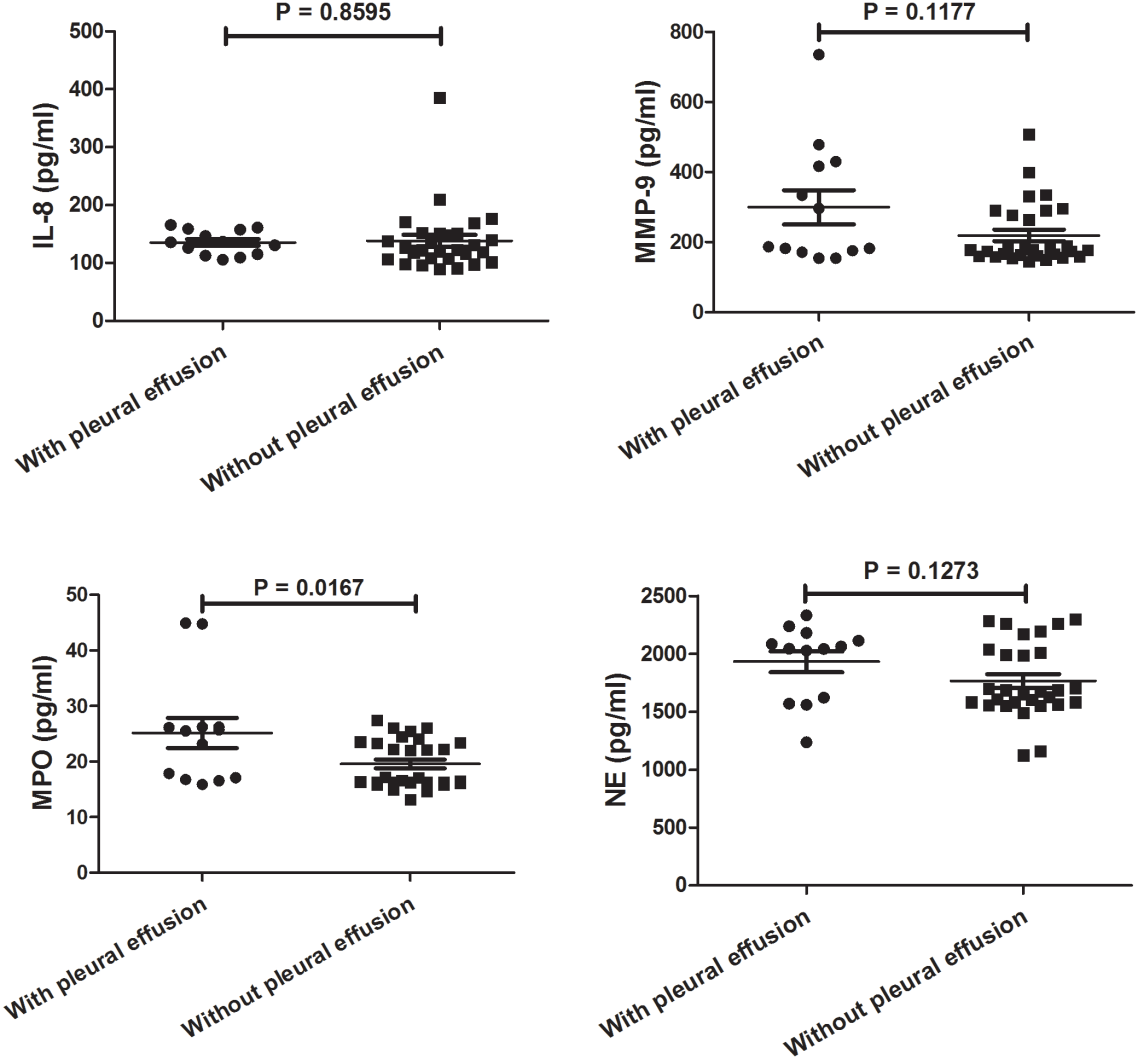
Comparison of IL-8, MPO, MMP-9, and NE in BALF between Patients with and without Pleural Effusion. The values in the graphs represent the mean ± SD. The P values were calculated using Student’s t test while Mann-Whitney U-test was used for comparison of MMP-9 and MPO between two groups. IL: interleukin-8; MPO: myeloperoxidase; MMP-9: matrix metalloproteinase 9; NE: neutrophil elastase.

Interestingly, the level of MPO in children with pleural effusion was significantly higher than in children without pleural effusion. No significant difference was found in IL-8, MMP-9, and NE between children with and without pleural effusion as shown in Fig 3.

### Relationships between IL-8, MPO, MMP-9, NE, and clinical profiles

The MPO level in children with MPP was associated with duration of fever (r = 0.332, P = 0.032) and MMP-9 level was associated with length of stay (r = 0.342, P = 0.026). No association was found between IL-8, MPO, MMP-9, NE, and other clinical aspects (P > 0.05). Because of interaction between MPO and NE (r = 0.472, P = 0.002), partial correlation was conducted and MPO was still associated with duration of fever (r = 0.353, P = 0.024).

### Levels of IL-8, MPO, MPP-9, and NE decreased after treatment

**Fig 4:**
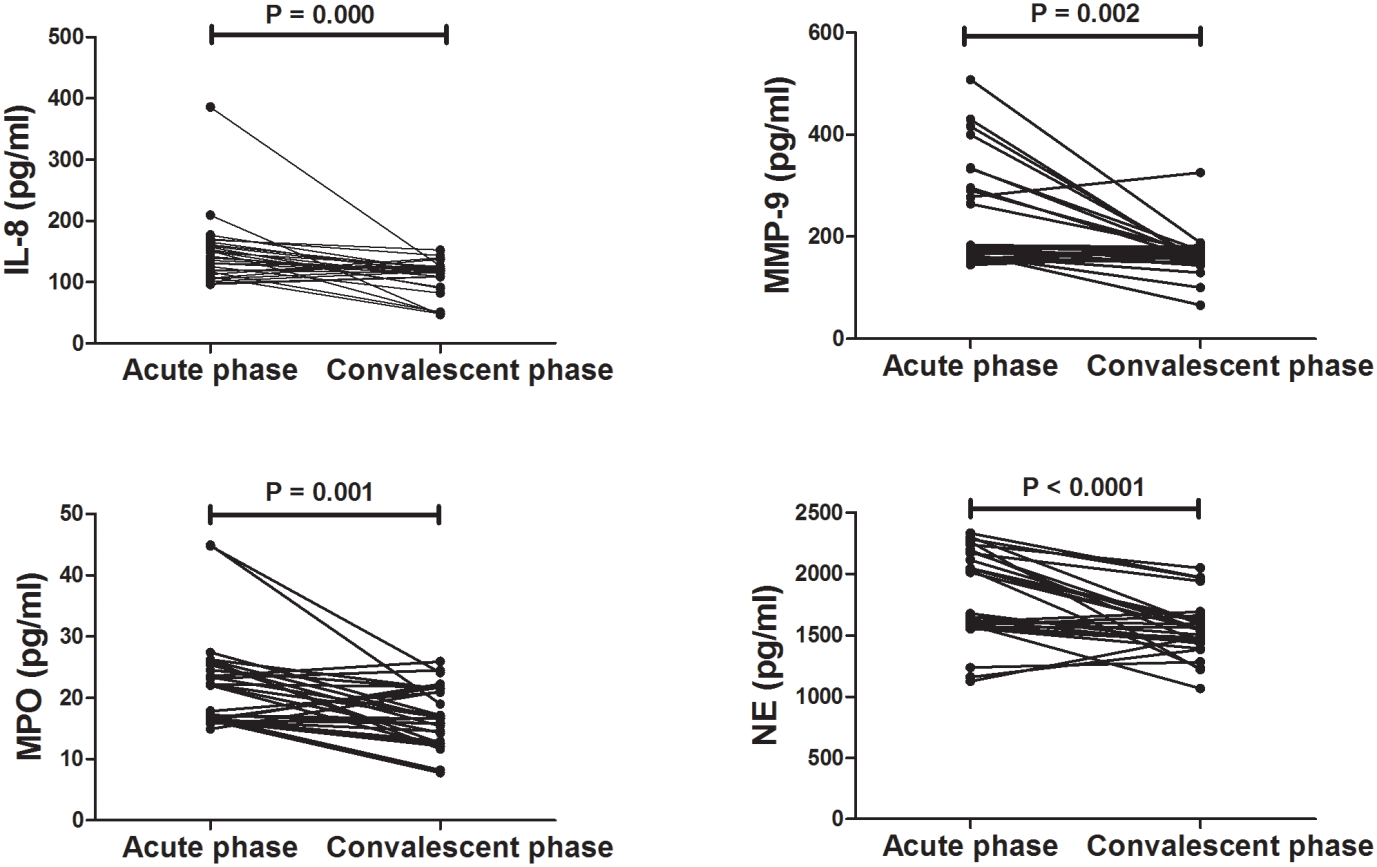
Comparison of IL-8, MPO, MMP-9, and NE in BALF before and after Treatment. The P values were calculated using Paired t test while Wilcoxon test was used for comparison of IL-8 and MMP-9 before and after treatment. IL: interleukin-8; MPO: myeloperoxidase; MMP-9: matrix metalloproteinase 9; NE: neutrophil elastase.

Convalescent BALF samples were obtained from 30 MPP patients and concentrations of IL-8, MPO, MPP-9, and NE significantly decreased after treatment shown in Fig 4.

### *M*. *pneumoniae* induced IL-8 release from NHBE

**Fig 5:**
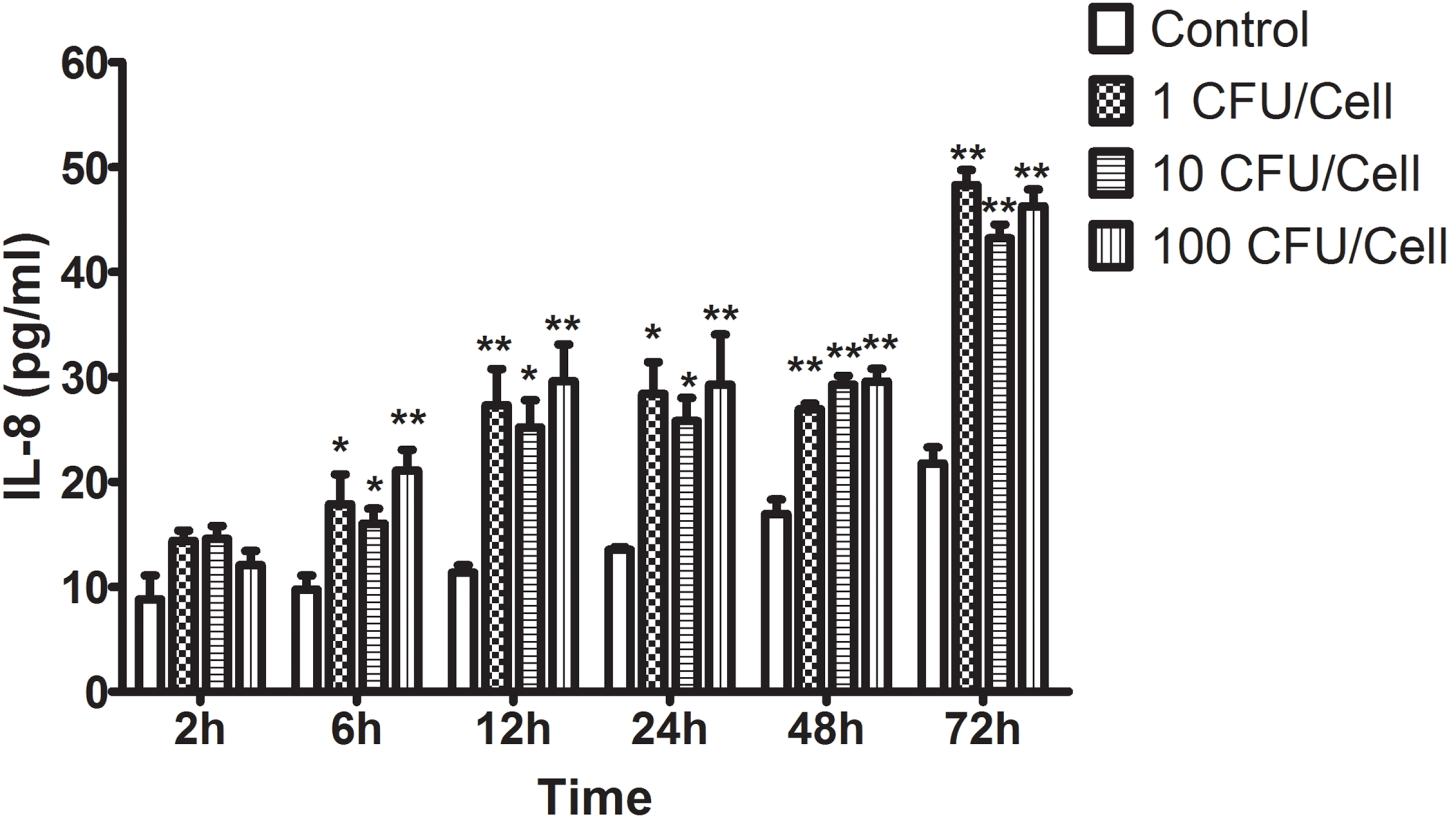
Concentrations of IL-8 Secreted by NHBE Cells after *M*. *pneumoniae* Infection. *M*. *pneumoniae* induced IL-8 production in a time-dependent manner. Data are represented as means ± SEMs from two independent experiments (* P < 0.05, ** P < 0.01 vs. controls). No significant difference was found between various multiplicities of infection (1–100 CFU/cell). The P values were calculated using ANOVA. IL: interleukin-8; NHBE: normal human bronchial epithelium.

*In vitro*, IL-8 release from NHBE was detected after *M*. *pneumoniae* stimulation. As seen in Fig 5, *M*. *pneumoniae* induced IL-8 production in a time-dependent manner in the 72-h period when compared with medium controls. However, there was no difference among various multiplicities of infection from 1 CFU/cell to 100 CFU/cell.

### Neutrophil activity induced by IL-8

**Fig 6:**
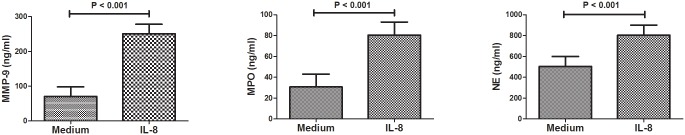
MPO, MMP-9, and NE Production Induced by Neutrophils after IL-8 Stimulation Significantly Increased Compared with Medium Controls. Data are represented as means ± SEMs. The P values were calculated using Student’s t test. IL-8: interleukin-8; MPO: myeloperoxidase; MMP-9: matrix metalloproteinase 9; NE: neutrophil elastase.

*In vitro*, neutrophil activity including MPO, MMP-9, and NE release was analyzed after IL-8 stimulation. As shown in Fig 6, concentrations of MPO, MMP-9, and NE in the supernatant significantly increased compared with medium controls.

## Discussion

Inflammation is a fundamental innate immune response to environmental factors, including infections. Excessive release of proinflammatory cytokines can occur following infection that skews the host response to "hyperinflammation" with exaggerated tissue damage. An excessive inflammation response induced by the host’s innate and adaptive immune systems is one of the main causes of the immunopathogenesis of *M*. *pneumoniae* infection and contributes to clinical presentations [11]. Various cells are involved in the inflammation response, such as macrophages, lymphocytes, bronchial epithelial cells as well as neutrophils. Macrophages rather than neutrophils are essential for the clearance of *M*. *pneumoniae* from the lungs [12]. On the contrary, neutrophil accumulation might lead to “hyperinflammation” due to MPO, MMP-9, and NE release.

The present study focused on the neutrophil activity induced by high expression of IL-8 and associations with clinical characteristics and laboratory findings. The findings are evidence that the *M*. *pneumoniae/*IL-8/neutrophil axis likely plays an important role in the pathogenesis of MPP based on both BALF analyses from children with MPP and experiments *in vitro*. We presumed that *M*. *pneumoniae* attaches to bronchial epithelial cells and induces the release of IL-8, which in turn drives the recruitment and activation of neutrophils.

However, our study did not show significance difference of IL-8 between patients with low *M*. *pneumoniae* DNA and high *M*. *pneumoniae* DNA, neither did show difference among various multiplicities of infection *in vitro*. There was no dose-response relationship between *M*. *pneumoniae* and IL-8 expression. Nevertheless, children with high *M*. *pneumoniae* load presented more neutrophils in BALF compared to children with low *M*. *pneumoniae* load. Taken together, other chemokines of neutrophils might take part in neutrophil accumulation in lungs.

Interestingly, MPO, MMP-9, and NE levels increased in the BALF of all patients with *M*. *pneumoniae* infection and decreased after treatment. MPO and MMP-9 might be effective biomarkers to predict disease severity in the present study. Previous studies have reported several biomarkers in serum or BALF such as soluble B7-H3 [13], IL-18 [14], MUC18 [15] as well as the community-acquired respiratory distress syndrome (CARDS) toxin which is an unique *M*. *pneumoniae* virulence factor regulating inflammasome activity [16, 17]. However, further studies in large, well-characterized patient samples are needed to confirm and explore the clinical applications of these observations.

IL-8 is a mediator between *M*. *pneumoniae* and neutrophils. It is reported that *M*. *pneumoniae* components (whole organism lysate or membrane extracts) could induce IL-8 release in the bronchial epithelium through ERK or NF-κB in a time and dose-dependent manner [18, 19]. Study showed NHBE cells infected with live *M*. *pneumoniae* might induce CARDS toxin production which causes IL-8 secretion [20]. IL-8 release could also be induced in macrophages by microbes [21]. Moreover, the *M*. *pneumoniae* extract could induce IL-17 release and subsequently cause neutrophil accumulation in the lung [22].

Meanwhile, IL-8 production by NHBE infected with *M*. *pneumoniae*, acts on neutrophils induce MPO, MMP-9, and NE release that leads to inflammation and tissue damage. A previous study showed that IL-8-induced MMP-9 release from neutrophils is mediated through CXCR2 and involves two distinct pathways, one involving PKC and ERK1/2 and the other involving Src-family kinases [23]. MPO and NE release in neutrophils stimulated by IL-8 from younger individuals significantly increased compared to medium controls [24].

However, some limitations of this study should be noted. First of all, this study only included 42 MPP cases which do not conform to a large samples study. Secondly, the data analysis alone may not serve as a conclusive interpretation because of lacking of the study for *M*. *pneumoniae* infection model *in Vivo*. What’s more, our study was based on a single center for data, which might have potential biases.

## Conclusion

Our study elucidates that bronchial epithelial cells infected by *M*. *pneumoniae* overexpressed IL-8, which subsequently enhanced neutrophils activity through MPO, MMP-9, and NE release. Consequently, the *M*. *Pneumoniae*/IL-8/neutrophil axis likely plays a vital role in the pathogenesis of MPP.

## Supporting Information

S1 Text Supporting data of tables and figures. (XLS)
